# The triose phosphate utilization limitation of photosynthetic rate: Out of global models but important for leaf models

**DOI:** 10.1111/pce.14153

**Published:** 2021-07-27

**Authors:** Luke M. Gregory, Alan M. McClain, David M. Kramer, Jeremy D. Pardo, Kaila E. Smith, Oliver L. Tessmer, Berkley J. Walker, Leonardo G. Ziccardi, Thomas D. Sharkey

**Affiliations:** ^1^ MSU‐DOE Plant Research Laboratory Michigan State University East Lansing Michigan USA; ^2^ Department of Plant Biology Michigan State University East Lansing Michigan USA; ^3^ Department of Biochemistry and Molecular Biology Michigan State University East Lansing Michigan USA; ^4^ Plant Biotechnology for Health and Sustainability Program Michigan State University East Lansing Michigan USA; ^5^ Department of Forestry Michigan State University East Lansing Michigan USA

## Abstract

The effect of including triose phosphate utilization (TPU) on parameterization of the Farquhar, von Caemmerer, Berry model of photosynthetic gas exchange measurements is explored. Better fits to data are found even though TPU rarely limits photosynthesis under physiological conditions.

Xiao et al. (2021) presented a method for assessing the variability of estimated parameters of the Farquhar, von Caemmerer, Berry (FvCB) model of photosynthesis (Farquhar et al. 1980). This model has been very effective at predicting photosynthetic responses to CO_2_, light and temperature, but estimating the parameters of the model can be difficult, with the fitted parameters having various degrees of uncertainty as demonstrated by Xiao et al. (2021). The original model assumed one of two conditions: (1) rubisco is saturated with ribulose 1, 5‐bisphosphate (RuBP) and so responds to CO_2_ with Michalis–Menten kinetics (rubisco‐limited) or (2) rubisco uses RuBP as fast as it is made (RuBP regeneration–limited). In condition (2), rubisco activity is determined by the rate of RuBP regeneration, typically as a result of being light‐limited. But even though photosynthetic CO_2_ assimilation (*A*) is light limited, it responds to increasing CO_2_ because of suppression of photorespiration by CO_2_. Carboxylation plus oxygenation stays constant under RuBP‐regeneration‐limited conditions, so if oxygenation goes down as CO_2_ increases, carboxylation will go up. The model was expanded to include a third condition, where RuBP regeneration is limited by how fast phosphorylated intermediates, primarily triose phosphates, are converted to end products, thereby releasing phosphate (Sharkey 1985). This is usually called “triose phosphate utilization (*TPU*) limitation.”

The FvCB model is most often parameterized by measuring *A* as a function of CO_2_ inside the air spaces of the leaf (*C*_*i*_), called an *A*/*C*_*i*_ curve. Rubisco‐limited assimilation shows a strong response to CO_2_, while RuBP‐regeneration–limited assimilation shows less response but still increases with increasing CO_2_. TPU‐limited points are characterized by no response to CO_2_ and, sometimes, an inhibition under increasing CO_2_ (Laporte et al. 2001). The condition is further diagnosed by a decline in photosynthetic electron transport caused by an increase in CO_2_ or decrease in O_2_, which can be measured by the chlorophyll fluorescence analysis (Sharkey et al. 1988). The TPU limitation is rarely seen at physiological CO_2_ partial pressure and temperature but is very frequently seen when CO_2_ is marginally higher than what the plant experienced during growth, especially if the temperature during the measurement is lower than the growth temperature (Sage & Sharkey 1987). Increasing the capacity for sucrose synthesis reduces the temperature at which *TPU* is observed (Laporte et al. 2001). *TPU* limitations are also associated with oscillations in photosynthetic rate (Sharkey et al. 1986), complicating measurements of *TPU*‐limited *A*.

The parameters that can be estimated by the fitting models are the maximum rate of rubisco carboxylation (*V*_cmax_) and the rate of electron transport (*J*) (since the analysis can be carried out at limiting light, this need not be *J*_max_). Also estimated are the respiration in the light (*R*_*L*_) (previously called day respiration, *R*_*d*_) and mesophyll conductance (*g*_*m*_). If *TPU* is considered, it is also estimated. We have used equations proposed by Busch et al. (2018) to include carbon flow out of photorespiration as glycine (*α*_*G*_) or serine (*α*_*S*_).

Some groups have concluded that TPU limitations are likely to be small and thus, constitute an unnecessary complication for modeling photosynthesis at global scales (Kumarathunge et al. 2019; Rogers et al. 2021). Moreover, there is evidence that when plants experience *TPU* for a sustained period, both rubisco capacity and electron transport capacity are reduced until *TPU* is no longer evident. Xiao et al. (2021) recently described Bayesian methods for estimating parameters of the FvCB model and the uncertainties in those estimates but without including *TPU* in their fitting. We have reanalysed the data of Xiao et al. (2021) to test the effect of inclusion of *TPU* on estimates of other parameters.

We began by re‐analysing the experimental data provided by Xiao et al. (2021). Four *A*/*C*_*i*_ curves measured with rice were provided. In three out of four cases, reverse sensitivity to CO_2_ of *A* was observed, and in all four cases, photochemical yield of photosystem II (Φ_II_) (measured by chlorophyll fluorescence analysis) declined at high CO_2_ (Figure 1). In repetition 2, Φ_II_ increased at low CO_2_ as rubisco activity increased then abruptly began to decline with increasing CO_2_, indicating a transition to *TPU* limitation with no points showing clear RuBP regeneration limitation (constant Φ_II_ with changing CO_2_).

**FIGURE 1:**
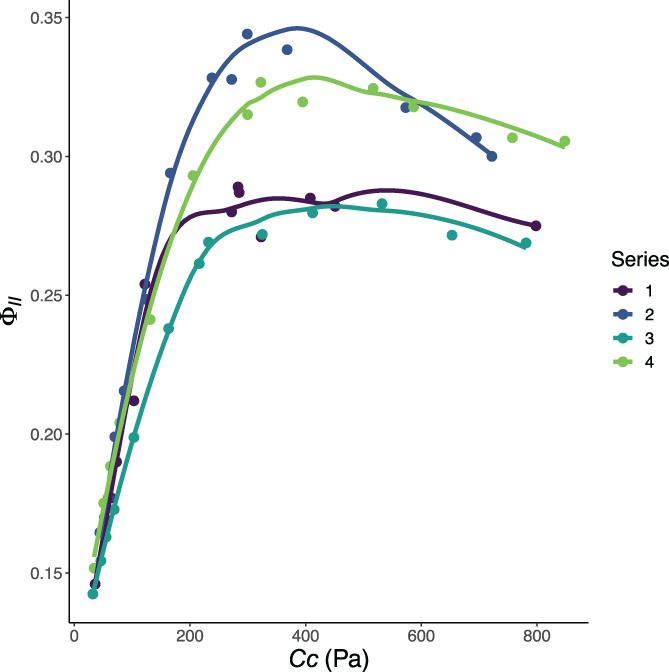
Φ_II_ values reported for the four replications of Xiao et al. (2021). Values were determined by chlorophyll fluorescence analysis. Curves 2 and 4 show an abrupt reversal from rubisco‐limited (Φ_II_ increasing with increasing CO_2_) to triose phosphate utilization *(TPU)*‐limited (Φ_II_ decreasing with increasing CO_2_) behaviour with no definitive RuBP regeneration limitation (Φ_II_ independent of changes in CO_2_) [Colour figure can be viewed at wileyonlinelibrary.com]

These behaviours indicate that *TPU* was occurring in all four repetitions. The authors specified in their methods section that they had to wait much longer for stability at high CO_2_ concentrations, and the data at high CO_2_ were noisy, also an indicator of *TPU*. Because TPU limitation is evident in the data, we tested the effect of adding *TPU* to the analysis.

We converted the most recent version (2.9) of the fitting spreadsheet that has been provided by Plant Cell and Environment (Sharkey 2016) to an R script with a user‐friendly interface (Shiny app), see https://github.com/poales/msuRACiFit.

The script iteratively fits data sets to biochemical models using rubisco‐limited, RuBP‐regeneration‐limited or *TPU*‐limited assumptions and, then, calculates which process is likely to be rate‐limiting for each data point, thus eliminating the need to assign specific limiting process to each of the data points.

We then fitted the data supplied by Xiao et al. (2021), first without *TPU* and then with *TPU* (Figure 2). For all four curves supplied, including *TPU* in the fitting improved the fit to the data at high CO_2_ and this was reflected in a reduction in the sum of the squared residuals (SSR), by 90% in three out of four repetitions (Table 1). The reduction in SSRs was much greater than what could be accounted for by the increase in degrees of freedom introduced by fitting additional parameters (i.e., *TPU*).

**FIGURE 2:**
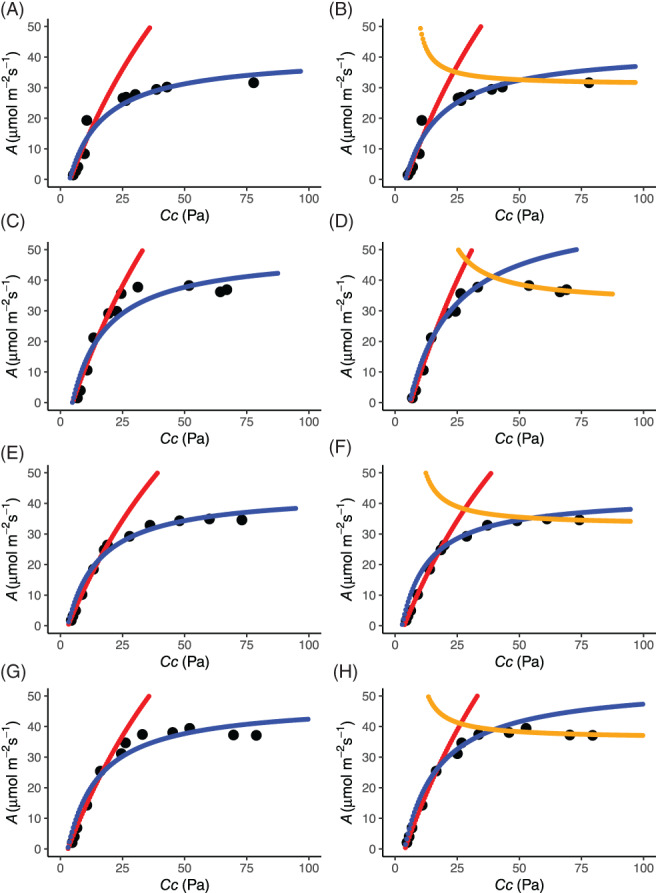
Fitting *A/C*_*i*_ curves. Fits to rice data (replications 1–4 of Xiao et al. 2021) without triose phosphate utilization *(TPU)* (A, C, E, G) or with *TPU* (B, D, F, H). Red is the fitted shape for rubisco‐limited condition, blue is for the RuBP regeneration–limited condition and gold is for the *TPU*‐limited condition [Colour figure can be viewed at wileyonlinelibrary.com]

**TABLE 1:** Comparisons of parameter values and sum of squared residuals (SSR)

		Rep 1		Rep 2		Rep 3		Rep 4	
	Units	without TPU	with TPU	without TPU	with TPU	without TPU	with TPU	without TPU	with TPU
*V*_*cmax*_	μmol m^−2^ s^−1^	183	194	203	232	167	174	179	197
*J*	μmol m^−2^ s^−1^	170	178	201	273	177	185	194	222
*TPU*	μmol m^−2^ s^−1^	–	10.9	–	12.3	–	12.1	–	12.4
*g*_*m*_	μmol m^−2^ s^−1^ pa^−1^	11.4	12.4	6.2	9.5	5.9	7.3	5.5	6.0
*R*_*L*_	μmol m^−2^ s^−1^	1.91	1.82	0.72	4.60	0.60	3.55	0.41	1.24
*a*_*G*_	Unitless	0.33	0.22	0.00	0.01	0.40	0.59	0.38	0.26
*a*_*S*_	Unitless	0.00	0.00	0.00	0.36	0.00	0.00	0.00	0.00
SSR	(μmol m^−2^ s^−1^)^2^	73.3	53.3	174.4	16.9	19.0	1.2	73.8	7.0

*Note*: Rice data of Xiao et al. (2021) was analysed with and without triose phosphate utilization *(TPU)* (fittings of the data in Figure 2 (A) ‐ (H)). *J* will always be underestimated when *TPU* limited points are treated as being *J*‐limited.

Abbreviations: SSR, sum of squared residuals; TPU, triose phosphate utilization.

When data points are treated as *J*‐limited but are actually limited by another process such as *TPU*, *J* will be underestimated. The estimate of *J* was higher when *TPU* was included in the analysis (Table 1), but if none of the points are definitely *J*‐limited (e.g., repetition 2), then the estimate of *J* is an estimate of the minimum *J*, not a true estimate of *J*. Because *J*‐limited measurements hold the most information concerning *g*_*m*_, *g*_*m*_ can be difficult to estimate when *A*/*C*_*i*_ curves are measured at saturating light. Using high but not saturating light can de‐emphasize *TPU* limitation and increase the amount of *J*‐limited data, which can improve estimates of *g*_*m*_ (Sharkey 2019; see box 1 of that paper). We also note that the method of splitting the measurement of the *A/C*_*i*_ curve, going from ambient down, returning to ambient and then going up, sometimes introduces noise that is especially apparent in the chlorophyll fluorescence data (see e.g., repetition 4, Figure 1 light green data and Figure 2 panels G and H). This noise in the data comes at the part of the curve that provides most information about *g*_*m*_, and so it is best to avoid the split method of measuring *A/C*_*i*_ curves.

We conclude that (a) it is important to include *TPU* when fitting *A*/*C*_*i*_ curves when there is evidence that *TPU* is occurring and (b) additional data may be needed depending on how the fittings are to be used, for example, it may be necessary to measure curves at saturating and also subsaturating light to get robust measures of all parameters. Because there are many parameters being fitted, some of which are complimentary, there is a danger of over fitting. When possible, parameters should be determined by independent measures. For example, *g*_*m*_ and *R*_*L*_ can be estimated independently and then fixed during fitting.

It must be accepted that some parameters can change within minutes, and this biological source of variance should be considered. Very rapid, monotonic *A/C*_*i*_ curves are likely to be very helpful in assessing the physiology of photosynthesis just as a high‐speed shutter on a camera avoids blurring an image, especially when the subject is dynamic. The latest technology released by LI‐COR allows *A/C*_*i*_ curves to be measured in under 5 minutes (https://www.licor.com/env/support/LI-6800/videos/dynamic-assimilation-technique.html).

Reporting the parameters of the FvCB model can be helpful for global modeling, for detecting effects of the environment on photosynthesis and changes in specific components of photosynthetic capacity. Because *TPU* is normally a temporary condition, inclusion in global models of photosynthesis may be an unnecessary complication of such models (Kumarathunge et al. 2019; Rogers et al. 2021). However, for laboratory studies or studies of initial effects of environmental changes on photosynthetic capacity, *TPU* is an important parameter to include in fitting routines, and significant uncertainties can arise when it is not included in analysis of *A/C*_*i*_ curves.

For large datasets, fitting batches of curves using programs like R can be very helpful. We supply an R package used in this work, together with a Shiny app for ease of fitting. What is presented expands on an earlier R Package (Duursma 2015). The Shiny app allows users to test specific hypotheses and can be a convenient way to explore how changing conditions such as temperature and light affect predicted rates of photosynthesis.

## CONFLICT OF INTEREST

The authors declare no known conflicts of interest.

## Data Availability

We used previously published data for this work and created an R script that is available. Please see https://github.com/poales/msuRACiFit for how to access and use the R script and Shiny app used for this work.
